# 4-(2-Carb­oxy­vin­yl)pyridinium iodide

**DOI:** 10.1107/S1600536810021501

**Published:** 2010-06-16

**Authors:** Dong-Yue Hu

**Affiliations:** a Ordered Matter Science Research Center, College of Chemistry and Chemical Engineering, Southeast University, Nanjing 210096, People’s Republic of China

## Abstract

In the crystal structure of the title salt, C_8_H_8_NO_2_^+^·I^−^, the cations and anions are linked by bifurcated N—H⋯(O,I) hydrogen bonds. A near-linear O—H⋯I hydrogen bond also exists between the cation and anion, resulting in a two-dimensional network. In the cation, the carboxyl group is twisted with respect to the pyridine ring at a dihedral angle of 15.34 (17)°.

## Related literature

3-(Pyridin-4-yl)acrylic acid is an inter­mediate in the synthesis of 3-amino-3-(pyridin-4-yl)propanoic acid, which is of inter­est as a precursor for the synthesis of novel biologically active compounds, see: Cohen *et al.* (2002 ▶); Qu *et al.* (2004 ▶).
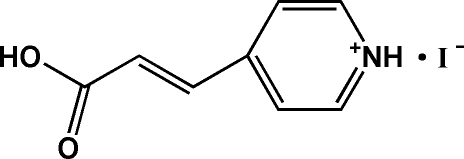


## Experimental

### 

#### Crystal data

C_8_H_8_NO_2_^+^·I^−^
*M* *_r_* = 277.05
Monoclinic, 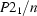

*a* = 4.9685 (10) Å
*b* = 15.494 (3) Å
*c* = 12.123 (2) Å
β = 101.48 (3)°
*V* = 914.6 (3) Å^3^
*Z* = 4
Mo *K*α radiation
μ = 3.46 mm^−1^
*T* = 293 K
0.20 × 0.20 × 0.20 mm

#### Data collection

Rigaku SCXmini diffractometer
Absorption correction: multi-scan (*CrystalClear*; Rigaku, 2005 ▶) *T*_min_ = 0.492, *T*_max_ = 0.518
9130 measured reflections
2099 independent reflections
1786 reflections with *I* > 2σ(*I*)
*R*_int_ = 0.046

#### Refinement

*R*[*F* ^2^ > 2σ(*F* ^2^)] = 0.028
*wR*(*F* ^2^) = 0.064
*S* = 1.11
2099 reflections
110 parameters
H-atom parameters constrained
Δρ_max_ = 0.54 e Å^−3^
Δρ_min_ = −0.49 e Å^−3^

### 

Data collection: *CrystalClear* (Rigaku, 2005 ▶); cell refinement: *CrystalClear*; data reduction: *CrystalClear*; program(s) used to solve structure: *SHELXS97* (Sheldrick, 2008 ▶); program(s) used to refine structure: *SHELXL97* (Sheldrick, 2008 ▶); molecular graphics: *SHELXTL* (Sheldrick, 2008 ▶); software used to prepare material for publication: *PRPKAPPA* (Ferguson, 1999 ▶).

## Supplementary Material

Crystal structure: contains datablocks I, global. DOI: 10.1107/S1600536810021501/xu2766sup1.cif

Structure factors: contains datablocks I. DOI: 10.1107/S1600536810021501/xu2766Isup2.hkl

Additional supplementary materials:  crystallographic information; 3D view; checkCIF report

## Figures and Tables

**Table 1:** Hydrogen-bond geometry (Å, °)

*D*—H⋯*A*	*D*—H	H⋯*A*	*D*⋯*A*	*D*—H⋯*A*
N1—H1⋯I1	0.86	3.04	3.652 (3)	130
N1—H1⋯O2^i^	0.86	2.15	2.819 (3)	134
O1—H1*B*⋯I1^ii^	0.82	2.54	3.362 (2)	175

Symmetry codes: (i) 
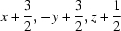
; (ii) 
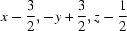
.

**Fig. 1.**
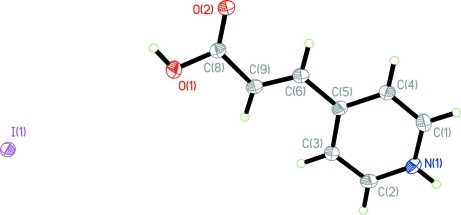
A partial packing diagram of the title compound, with the displacement ellipsoids were drawn at the 30% probability level.

**Fig. 2.**
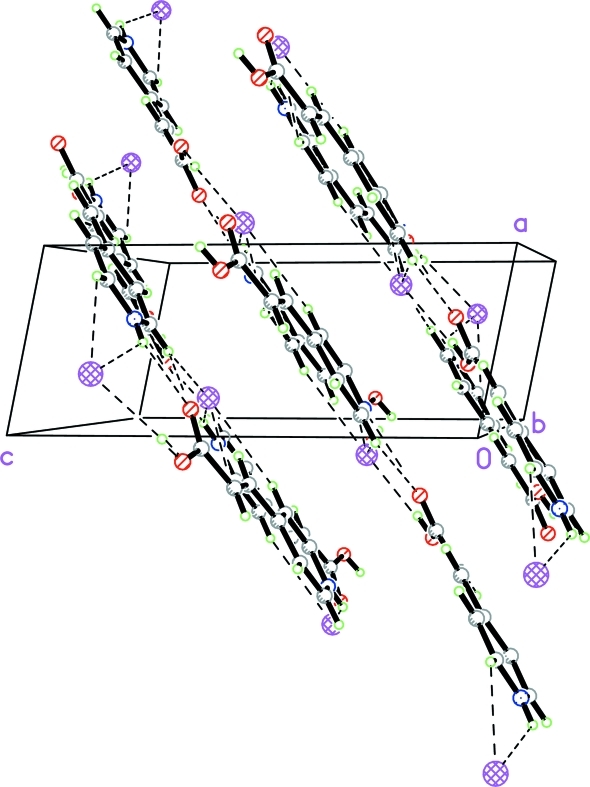
Packing diagram of the title compound, showing the structure along the b axis. Hydrogen bonds are shown as dashed lines.

## supplementary crystallographic information

### Comment

β-Amino acids are important molecules due to their pharmacological properties. Recently, there has been an increased interest in the enantiomeric preparation of β-amino acids as precursors for the synthesis of novel biologically active compounds (Cohen *et al.*, 2002; Qu *et al.*, 2004). 3-(Pyridin-4-yl)acrylic acid is the intermediate to synthesize 3-amino-3-(pyridin-4-yl)propanoic acid.

The asymmetric unit of the title compound (Fig. 1) contains one 4-(2-carboxyvinyl) pyridinium and one iodate anion. The conformation of the cation is stabilized by an intramolecular N—H···I and C—H···O hydrogen bond (Table 1). In the crystal structure (Fig. 2), molecules are connected by intermolecular N—H···O, O—H···I and C—H···O hydrogen bonds into chains running parallel to the *b* axis (Table 1).

The dielectric constant of the title compound as a function of temperature indicates that the permittivity is basically temperature-independent, suggesting that this compound should be not a real ferroelectrics or there may be no distinct phase transition occurred within the measured temperature range.

### Experimental

In a dry, N_2_-filled three-necked flask fitted with stirrer, 4-pyridinecarboxaldehyde (1.07 g, 10 mmol) and malonic acid (2.50 g, 24 mmol) were dissolved in pyridine (4 ml) and piperidine (0.1 ml) and this solution was refluxed for 4.5 h and the mixture was then worked up. To the suspension was then added ethylether (5 ml), and the white precipitate was filtered and washed with ethylether (3.5 ml) to give (E)-3-(4-pyridyl)acrylic acid. (E)-3-(4-pyridyl)acrylic acid (0.5 g, 3 mmol) and hydriodic acid (0.43 g, 3 mmol) were dissolved in ethanol (10 ml). After slow evaporation of the solution over a period of 3 days, orange prismatic crystals of the title compound suitable for X-ray diffraction analysis were isolated.

### Refinement

All H atoms were placed at calculated positions with C—H = 0.93, N—H = 0.86 and O—H = 0.82 Å, and refined in riding mode with U_iso_(H) = 1.5U_eq_(O) and 1.2U_eq_(C,N).

### Crystal data

**Table:**

C_8_H_8_NO_2_^+^·I^−^	*F*(000) = 528.0
*M**_r_* = 277.05	*D*_x_ = 2.012 Mg m^−^^3^
Monoclinic, *P*2_1_/*n*	Mo *K*α radiation, λ = 0.71073 Å
Hall symbol: -P 2yn	Cell parameters from 1866 reflections
*a* = 4.9685 (10) Å	θ = 3.2–27.0°
*b* = 15.494 (3) Å	µ = 3.46 mm^−^^1^
*c* = 12.123 (2) Å	*T* = 293 K
β = 101.48 (3)°	Prism, orange
*V* = 914.6 (3) Å^3^	0.20 × 0.20 × 0.20 mm
*Z* = 4	

### Data collection

**Table:**

Rigaku SCXmini diffractometer	2101 independent reflections
Radiation source: fine-focus sealed tube	1786 reflections with *I* > 2σ(*I*)
graphite	*R*_int_ = 0.046
Detector resolution: 13.6612 pixels mm^-1^	θ_max_ = 27.5°, θ_min_ = 3.1°
ω scan	*h* = −6→6
Absorption correction: multi-scan (*CrystalClear*; Rigaku, 2005)	*k* = −20→20
*T*_min_ = 0.492, *T*_max_ = 0.518	*l* = −15→15
9130 measured reflections	

### Refinement

**Table:**

Refinement on *F*^2^	Primary atom site location: structure-invariant direct methods
Least-squares matrix: full	Secondary atom site location: difference Fourier map
*R*[*F*^2^ > 2σ(*F*^2^)] = 0.028	Hydrogen site location: inferred from neighbouring sites
*wR*(*F*^2^) = 0.064	H-atom parameters constrained
*S* = 1.11	*w* = 1/[σ^2^(*F*_o_^2^) + (0.0215*P*)^2^ + 0.0886*P*] where *P* = (*F*_o_^2^ + 2*F*_c_^2^)/3
2099 reflections	(Δ/σ)_max_ = 0.001
110 parameters	Δρ_max_ = 0.54 e Å^−^^3^
0 restraints	Δρ_min_ = −0.49 e Å^−^^3^

### Special details

**Table:**

Geometry. All e.s.d.'s (except the e.s.d. in the dihedral angle between two l.s. planes) are estimated using the full covariance matrix. The cell e.s.d.'s are taken into account individually in the estimation of e.s.d.'s in distances, angles and torsion angles; correlations between e.s.d.'s in cell parameters are only used when they are defined by crystal symmetry. An approximate (isotropic) treatment of cell e.s.d.'s is used for estimating e.s.d.'s involving l.s. planes.
Refinement. Refinement of *F*^2^ against ALL reflections. The weighted *R*-factor *wR* and goodness of fit *S* are based on *F*^2^, conventional *R*-factors *R* are based on *F*, with *F* set to zero for negative *F*^2^. The threshold expression of *F*^2^ > σ(*F*^2^) is used only for calculating *R*-factors(gt) *etc*. and is not relevant to the choice of reflections for refinement. *R*-factors based on *F*^2^ are statistically about twice as large as those based on *F*, and *R*- factors based on ALL data will be even larger.

### Fractional atomic coordinates and isotropic or equivalent isotropic displacement parameters (Å^2^)

**Table:**

	*x*	*y*	*z*	*U*_iso_*/*U*_eq_	
N1	0.8838 (5)	0.62959 (16)	0.6895 (2)	0.0397 (6)	
H1	1.0091	0.5971	0.7275	0.048*	
C8	0.0739 (7)	0.91308 (18)	0.4041 (3)	0.0371 (7)	
C3	0.7164 (6)	0.76753 (19)	0.6401 (3)	0.0387 (7)	
H3	0.7325	0.8271	0.6486	0.046*	
C6	0.2864 (6)	0.78463 (18)	0.4944 (3)	0.0362 (7)	
H6	0.1361	0.7556	0.4527	0.043*	
C4	0.4734 (6)	0.64341 (19)	0.5602 (3)	0.0396 (8)	
H4	0.3229	0.6179	0.5138	0.048*	
C5	0.4953 (6)	0.73228 (18)	0.5661 (2)	0.0321 (6)	
C2	0.9097 (6)	0.7147 (2)	0.7004 (3)	0.0423 (8)	
H2	1.0602	0.7382	0.7492	0.051*	
C1	0.6722 (7)	0.59325 (19)	0.6224 (3)	0.0447 (8)	
H1A	0.6590	0.5334	0.6176	0.054*	
O1	0.1334 (5)	0.99482 (13)	0.3899 (2)	0.0527 (7)	
H1B	0.0129	1.0164	0.3417	0.079*	
O2	−0.1307 (4)	0.87875 (15)	0.3553 (2)	0.0545 (7)	
C9	0.2913 (6)	0.8691 (2)	0.4834 (3)	0.0377 (7)	
H9	0.4334	0.9009	0.5262	0.045*	
I1	1.17185 (4)	0.413232 (12)	0.682811 (18)	0.04391 (10)	

### Atomic displacement parameters (Å^2^)

**Table:**

	*U*^11^	*U*^22^	*U*^33^	*U*^12^	*U*^13^	*U*^23^
N1	0.0317 (13)	0.0381 (14)	0.0457 (16)	0.0063 (12)	−0.0011 (12)	0.0076 (12)
C8	0.0352 (17)	0.0361 (17)	0.0362 (18)	0.0011 (13)	−0.0017 (14)	0.0004 (13)
C3	0.0338 (16)	0.0316 (15)	0.0467 (18)	−0.0007 (13)	−0.0019 (13)	−0.0005 (14)
C6	0.0326 (16)	0.0378 (16)	0.0348 (17)	−0.0021 (13)	−0.0019 (13)	−0.0013 (13)
C4	0.0345 (17)	0.0370 (16)	0.0425 (19)	−0.0007 (13)	−0.0042 (14)	−0.0072 (14)
C5	0.0296 (14)	0.0357 (15)	0.0290 (16)	0.0020 (12)	0.0011 (12)	0.0011 (12)
C2	0.0324 (16)	0.0444 (18)	0.044 (2)	−0.0041 (14)	−0.0058 (14)	0.0001 (15)
C1	0.047 (2)	0.0318 (17)	0.052 (2)	−0.0002 (14)	0.0021 (17)	0.0012 (14)
O1	0.0523 (15)	0.0370 (12)	0.0579 (16)	−0.0062 (11)	−0.0152 (12)	0.0125 (11)
O2	0.0441 (14)	0.0390 (12)	0.0663 (17)	−0.0051 (11)	−0.0227 (12)	0.0069 (12)
C9	0.0330 (16)	0.0394 (17)	0.0350 (17)	−0.0017 (13)	−0.0067 (13)	0.0005 (13)
I1	0.04248 (15)	0.03397 (14)	0.04898 (17)	−0.00119 (9)	−0.00609 (11)	−0.00440 (9)

### Geometric parameters (Å, °)

**Table:**

N1—C1	1.321 (4)	C6—C5	1.460 (4)
N1—C2	1.330 (4)	C6—H6	0.9300
N1—H1	0.8600	C4—C1	1.360 (4)
C8—O2	1.195 (4)	C4—C5	1.382 (4)
C8—O1	1.319 (3)	C4—H4	0.9300
C8—C9	1.464 (4)	C2—H2	0.9300
C3—C2	1.359 (4)	C1—H1A	0.9300
C3—C5	1.385 (4)	O1—H1B	0.8200
C3—H3	0.9300	C9—H9	0.9300
C6—C9	1.316 (4)		
			
C1—N1—C2	122.3 (3)	C5—C4—H4	120.0
C1—N1—H1	118.9	C4—C5—C3	118.0 (3)
C2—N1—H1	118.9	C4—C5—C6	118.9 (3)
O2—C8—O1	123.6 (3)	C3—C5—C6	123.0 (3)
O2—C8—C9	124.2 (3)	N1—C2—C3	120.0 (3)
O1—C8—C9	112.2 (3)	N1—C2—H2	120.0
C2—C3—C5	119.7 (3)	C3—C2—H2	120.0
C2—C3—H3	120.1	N1—C1—C4	119.9 (3)
C5—C3—H3	120.1	N1—C1—H1A	120.0
C9—C6—C5	126.0 (3)	C4—C1—H1A	120.0
C9—C6—H6	117.0	C8—O1—H1B	109.5
C5—C6—H6	117.0	C6—C9—C8	120.2 (3)
C1—C4—C5	120.0 (3)	C6—C9—H9	119.9
C1—C4—H4	120.0	C8—C9—H9	119.9

### Hydrogen-bond geometry (Å, °)

**Table:**

*D*—H···*A*	*D*—H	H···*A*	*D*···*A*	*D*—H···*A*
N1—H1···I1	0.86	3.04	3.652 (3)	130
N1—H1···O2^i^	0.86	2.15	2.819 (3)	134
O1—H1B···I1^ii^	0.82	2.54	3.362 (2)	175

Symmetry codes: (i) *x*+3/2, −*y*+3/2, *z*+1/2; (ii) *x*−3/2, −*y*+3/2, *z*−1/2.

## References

[bb1] Cohen, J. H., Abdel-Magid, A. F., Almond, H. R. Jr & Maryanoff, C. A. (2002). *Tetrahedron Lett.* **43**, 1977–1981.

[bb2] Ferguson, G. (1999). *PRPKAPPA* University of Guelph, Canada.

[bb3] Qu, Z.-R., Zhao, H., Wang, Y.-P., Wang, X.-S., Ye, Q., Li, Y.-H., Xiong, R.-G., Abrahams, B. F., Liu, Z.-G. & Xue, Z.-L. (2004). *Chem. Eur. J.* **10**, 54–60.

[bb4] Rigaku (2005). *CrystalClear.* Rigaku Corporation, Tokyo, Japan.

[bb5] Sheldrick, G. M. (2008). *Acta Cryst.* A**64**, 112–122. 10.1107/S0108767307043930 18156677

